# Gill transcriptome response to changes in environmental calcium in the green spotted puffer fish

**DOI:** 10.1186/1471-2164-11-476

**Published:** 2010-08-17

**Authors:** Patrícia IS Pinto, Hideo Matsumura, Michael AS Thorne, Deborah M Power, Ryohei Terauchi, Richard Reinhardt, Adelino VM Canário

**Affiliations:** 1 Centro de Ciências do Mar (CCMAR), CIMAR-Laboratório Associado, University of Algarve, Campus de Gambelas, 8005-139 Faro, Portugal; 2 Gene Research Center, Shinshu University, Nagano, 390-8621, Japan; 3 British Antarctic Survey (BAS), High Cross, Madingley Road, Cambridge, CB3 0ET, UK; 4 Iwate Biotechnology Research Center (IBRC), 22-174-4 Narita, Kitakami, Iwate 024-0003, Japan; 5 Max Planck Institute for Molecular Genetics (MPIMG), Ihnestraße 63-73 - 14195 Berlin, Germany

## Abstract

**Background:**

Calcium ion is tightly regulated in body fluids and for euryhaline fish, which are exposed to rapid changes in environmental [Ca^2+^], homeostasis is especially challenging. The gill is the main organ of active calcium uptake and therefore plays a crucial role in the maintenance of calcium ion homeostasis. To study the molecular basis of the short-term responses to changing calcium availability, the whole gill transcriptome obtained by Super Serial Analysis of Gene Expression (SuperSAGE) of the euryhaline teleost green spotted puffer fish, *Tetraodon nigroviridis*, exposed to water with altered [Ca^2+^] was analysed.

**Results:**

Transfer of *T. nigroviridis *from 10 ppt water salinity containing 2.9 mM Ca^2+ ^to high (10 mM Ca^2+ ^) and low (0.01 mM Ca^2+^) calcium water of similar salinity for 2-12 h resulted in 1,339 differentially expressed SuperSAGE tags (26-bp transcript identifiers) in gills. Of these 869 tags (65%) were mapped to *T. nigroviridis *cDNAs or genomic DNA and 497 (57%) were assigned to known proteins. Thirteen percent of the genes matched multiple tags indicating alternative RNA transcripts. The main enriched gene ontology groups belong to Ca^2+ ^signaling/homeostasis but also muscle contraction, cytoskeleton, energy production/homeostasis and tissue remodeling. *K*-means clustering identified co-expressed transcripts with distinct patterns in response to water [Ca^2+^] and exposure time.

**Conclusions:**

The generated transcript expression patterns provide a framework of novel water calcium-responsive genes in the gill during the initial response after transfer to different [Ca^2+^]. This molecular response entails initial perception of alterations, activation of signaling networks and effectors and suggests active remodeling of cytoskeletal proteins during the initial acclimation process. Genes related to energy production and energy homeostasis are also up-regulated, probably reflecting the increased energetic needs of the acclimation response. This study is the first genome-wide transcriptome analysis of fish gills and is an important resource for future research on the short-term mechanisms involved in the gill acclimation responses to environmental Ca^2+ ^changes and osmoregulation.

## Background

Calcium (Ca^2+^) is a major component of the skeleton in vertebrates and the exoskeleton of some invertebrates (e.g. in bones, scales or shellfish shells) and plays key roles in a wide range of physiological processes, such as muscular contraction, modulation of permeability and excitability of plasma membranes, nerve signal transduction or intracellular signaling [1].

Terrestrial vertebrates obtain calcium mainly through their diet and whole-body calcium homeostasis is mainly achieved by intestinal absorption and kidney reabsorption [2]. The range of calcium in water can range from trace levels in soft fresh water to 10 mM in seawater and fish that live in those waters are able to retrieve most or all the calcium they need from the surrounding water. Furthermore, many species (e.g. estuarine) have the capacity to rapidly adjust to a wide range of environmental calcium concentrations [3]. The ability of teleost fishes to maintain circulating calcium [Ca^2+^] within narrow limits is well documented [4-6] and is mediated by rapid rate changes of water and calcium exchange in gill, intestine, kidney and skin epithelia [reviewed in [3]].

The gill epithelium is the main site of active Ca^2+ ^uptake from the water at low [Ca^2+^], while at higher [Ca^2+^], such as in seawater (SW), the intestine acquires also an important role [3,7]. The gill, together with the kidney, also appears to play a role in Ca^2+ ^excretion but little is known about this process [3,8]. Branchial Ca^2+ ^uptake appears to occur mainly through specialized mitochondrion-rich cells (MR cells) or "chloride cells" following a 3-step process similar to that proposed for Ca^2+ ^reabsorption in the mammalian kidney: passive entry of Ca^2+ ^through apical epithelial Ca^2+ ^channels (e.g. ECaC); transcellular transport in the cytoplasm or sequestration in organelles through binding to Ca^2+^-binding proteins (CaBPs); and active basolateral extrusion to the blood via a plasma membrane Ca^2+^-ATPase (PMCA) or a Na^+^/Ca^2+^-exchanger (NCX) [3,9]. When fish are exposed to low or high calcium environments, they respond by modifying gill Ca^2+ ^uptake; altering the number, area and morphology of MR cells, gene expression of epithelial calcium channel (*ecac*) and other osmoregulatory mediators [10-13]. However, there is still relatively little knowledge of the gill molecular machinery involved in the adjustments to changes in [Ca^2+^], in particular the early events.

Finally, the internal and external Ca^2+ ^sensing mechanisms (e.g. via a membrane calcium-sensing receptor, CaSR), intracellular signaling and the endocrine control of Ca^2+ ^balance in fish, which differs from that of terrestrial animals, have been the subject of several physiological studies and recent reviews [3,14-17], but much remains to be discovered about their mechanisms of action and target genes in Ca^2+^-transporting epithelia.

This study sought to detail the cellular mechanisms and determine the genes involved in the rapid perception, signal transduction and effector responses of teleost fish gills to changes in environmental Ca^2+^. To this end, we analyzed the gill transcriptome of the green spotted puffer fish (*Tetraodon nigroviridis*) soon after transfer from brackish water (10 ppt salinity, 2.9 mM Ca^2+^) to brackish water containing 0.01 and 10 mM Ca^2+^. The [Ca^2+^] tested cover the range teleost fish are generally exposed to in nature: from 0.01 mM in ion-poor fresh water to 10 mM in sea water [3]. The choice of time points analyzed after transfer (2 and 12 h) sought to capture the early transcriptomic responses of the acclimation process. *T. nigroviridis *was chosen as the experimental model because of two main reasons: 1) it is euryhaline and able to tolerate wide variations in water [Ca^2+^] (can be found in freshwater (FW) streams and rivers, estuaries and coastal waters [18] and in experimental conditions tolerate direct transfers from FW to SW and vice-versa [19]), and 2) its genome and many cDNAs have been sequenced [20], facilitating gene identification from sequenced data. The gill transcriptomic responses were obtained using a variation of serial analysis of gene expression (SAGE) [21] designated SuperSAGE, a global and quantitative transcriptomic technique based on high throughput sequencing and counting of small transcript-identifier tags, 26-bp instead of 14-bp in SAGE. The larger SuperSAGE tags results in improvements in tag-to-gene annotation (gene identification) and characterization of identified tags [22,23], and has been adapted to massively parallel pyrosequencing using a bar-code system which simplifies the procedure and permits higher throughput [24].

As a result of transfer of *T. nigroviridis *from water containing 2.9 mM Ca^2+ ^to water containing 0.01 and 10 mM Ca^2+ ^1,339 differentially expressed gill transcripts were identified and clustered into sub-groups of genes with distinct patterns of expression. Gene ontology (GO) enrichment analysis of the annotated tags allowed the identification of biological processes, functions and cell compartments that were most affected. Relative expression changes in 8 genes were also compared by quantitative PCR (qPCR). The data obtained in this study provide the first comprehensive catalogue of genes implicated in the rapid acclimation responses of fish gill cells to changes in water Ca^2+ ^availability.

## Results

### Total plasma calcium and Ca^2+ ^channel gene expression

**Figure 1:**
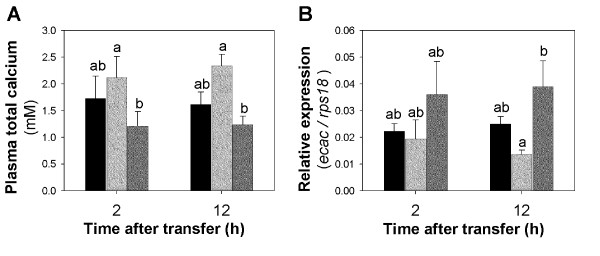
**Blood plasma total calcium and *ecac *gill mRNA expression upon exposure to different water [Ca^2+^]**. Each bar is the mean ± S.E.M of (A) the plasma total calcium levels (mM) and (B) the relative expression of the epithelial calcium channel mRNA in the gills (*ecac *expression quantified by qPCR and normalized to the reference gene *rps18*) of *T. nigroviridis *individuals (n = 5-6/group) after 2 or 12 h transfer to 10 ppt artificial water containing 2.9 mM Ca^2+ ^(control water, black bars), 10 mM Ca^2+ ^(HighCa water, light grey bars) or 0.01 mM Ca^2+ ^(LowCa water, dark grey bars). Different letters indicate statistically significant differences (p < 0.05) between groups, evaluated by two-way ANOVA.

Transfer of *T. nigroviridis *from water containing 2.9 mM Ca^2+ ^to water containing 0.01 (LowCa) and 10 mM Ca^2+ ^(HighCa) resulted, respectively, in a small decrease and increase in blood plasma total [Ca^2+^], both at 2 and 12 h, which was significantly different between the two experimental groups (Figure 1). At the same time the expression level of gill epithelial calcium channel mRNA (*ecac*, official name *trpv6*) changed in the opposite direction, i.e., up-regulation in LowCa and down-regulation in HighCa which were statistically significant (P < 0.05) between these experimental groups 12 h after transfer. The expression of CaSR transcripts was also detected at very low levels in the gills of all experimental groups by quantitative real-time (q) PCR (and by SuperSAGE in the LowCa2 h library, one tag with count 1), but no changes in gene expression in response to water [Ca^2+^] were detected (not shown). These results are in agreement with previous studies [10-13,25] and confirmed that the experimental approach induced a challenge to calcium balance mechanisms in *T. nigroviridis *gills.

### Effects on global gene expression

Five gill SuperSAGE libraries from control fish at 2 h (C2 h) and 12 h (C12 h), from LowCa at 2 h (LowCa2 h) and 12 h (LowCa12 h) and from HighCa at 12 h only (HighC12 h) were sequenced in a single half run of 454 pyrosequencing and yielded 302,033 reads (ditag sequences). Stringent quality control of ditag sequences yielded 344,375 26 bp-SuperSAGE mono-tags which were retained for further analysis (Table 1). Each library contained on average of ~69,000 tags of which ~26,500 were unique (unitags, corresponding to unique transcripts; Table 1).

**Table 1:** Summary of tag extraction from the *T. nigroviridis *gill SuperSAGE libraries

Library	C2 h	LowCa 2 h	C12 h	HighCa 12 h	LowCa 12 h	Total	Average	**% of total (average) **^**4**^
**Tag extraction from sequencing data**								
Total extracted tags ^1^	65,378	87,388	67,996	60,142	63,471	344,375	68,875	
# unitags ^2^	24,536	31,907	27,403	24,427	24,886		26,632	
% unitags	37.5	36.5	40.3	40.6	39.2		38.7	
								
**Distribution of Unitags by abundance classes ^3^**								
1	16,630	22,087	19,049	17,047	17,218	92,031	18,406	69.1
2 to 4	5,516	6,891	5,886	5,304	5,359	28,956	5,791	21.7
5 to 9	1,503	1,830	1,618	1,330	1,487	7,768	1,554	5.8
10 to 100	852	1,043	819	717	793	4,224	845	3.2
>100	35	56	31	29	29	180	36	0.1

^1^Total number of tags extracted from each library after quality selection. ^2 ^Unique tags (or Unitags, corresponding to unique transcripts) obtained for each library. ^3 ^Number of Unitags in each abundance class, based on their tag count in each library; ^4 ^Percentage of Unitags in each abundance category relative to the total number of Unitags per library (on average).

SuperSAGE libraries prepared from gills in control water for 2 or 12 h (C2 h and C12 h); low calcium water for 2 or 12 h (LowCa2 h and LowCa12 h) or high calcium water for 12 h (HighCa12 h).

Approximately 70% of the unitags were singletons (tags with 1 count), while the number of unique transcripts decreased for classes with higher abundance (Table 1). Singleton tags were included in the statistical analysis [26] to avoid losing information from low abundance transcripts, but those appearing only in one library (probably derived from sequencing errors) failed the stringent multiple tests to assess differential expression.

In order to eliminate genes potentially responding to handling stress (given the relatively brief duration of experimental exposure), the gill expression profiles of Ca^2+ ^challenged libraries were always compared to the gill profile of fish transferred to control water at the corresponding time-point (control libraries), while two types of statistical tests and multiple library comparisons (see methods) were also carried out to increase stringency and to account for time-effects. A total of 1,426 unique tags (transcripts) showing differential expression between control and [Ca^2+^] treatments were identified of which 1,339 were up- or down-regulated at least 2-fold and selected for further analyses.

### Tag annotation

The annotation of the differentially expressed tags was carried out by BlastN mapping to available DNA sequences for this species, followed by BlastX assignment of these longer (tag-matching) cDNAs or 1000-bp DNA fragments (extracted upstream from the tag-matching sites in the genome) to the highly curated non-redundant Swiss-Prot protein database.

In the BlastN step, carried out under high stringency (26/26 nucleotide matches), 516 tags (38.5%) matched to one or more cDNAs available in GenBank (NCBI cDNAs), 243 tags (18.1%) matched cDNAs predicted from the *T.nigroviridis *genome (Ensembl cDNAs) and 692 tags (51.7%) matched the genome (Additional file 1), making a total of 869 tags with a DNA match to available *T.nigroviridis *DNA datasets (Table 2). Three hundred and fifty three tags (26.4%) mapped only to the genome or Ensembl genome predicted cDNAs (Additional file 1) and may represent novel transcripts. It was not possible to calculate the rate of unambiguous tag mapping to unique transcripts, since the available cDNA dataset contained multiple redundant expressed sequence tags (ESTs) and no unigene [27] dataset is available for *T.nigroviridis*. However, only a reduced number of tags matched more than one location in the genome (28 tags, 4%), suggesting unambiguous annotation for most 26-bp tags.

**Table 2:** Summary of tag annotation of the 1,339 differentially expressed tags

	# tags	%
**BlastN summary **^**1**^		
Unitags with significant BlastN match	869	64.9
Unitags with no significant BlastN match	470	35.1
Total	1,339	100.0
		

**BlastX summary **^**2**^		
DNAs with significant BlastX match	497	57.2
DNAs with no significant BlastX match	372	42.8
Total	869	100.0
		
**Global Blast summary**		
Non-annotated Unitags	470	35.1
Unitags with match to anonymous DNAs	372	27.8
Unitags matching Swiss-Prot proteins	497	37.1
Total	1,339	100.0

^1^Total number (#) and percentage (%) of differentially expressed unique tags (Unitags) with a significant (26/26 nucleotides) or non significant BlastN match to nucleotide sequences of *T.nigroviridis *available in databases. ^2 ^Number and percentage of tag-matching *T.nigroviridis *cDNAs or genome fragments showing significant (E value < 10^-5^) or non-significant matches with the Swiss-Prot database. ^3 ^Summary of the 2-step tag annotation results (tag-to-DNA BlastN followed by DNA-to-protein BlastX).

Four hundred and seventy differentially expressed tags (35.1%) could not be mapped to any available DNA sequence and may represent genes or splice variant transcripts not previously covered by the sequencing of genomic and expressed sequence tag (EST) data. Depending on the DNA dataset, lowering stringency to 24/24 nucleotide matches improved annotation up to 19% (Additional file 2), suggesting that some tags contain nucleotide mismatches to available DNAs, potentially derived from sequencing errors or single-nucleotide polymorphisms (SNPs) in transcript sequences. However, even in these circumstances 60% (279) of the non-annotated tags could not be assigned, supporting the notion that they are not represented in available sequence databases. Therefore the 26/26 (perfect match) stringency rule was maintained in this analysis to ensure a less ambiguous annotation of differentially expressed tags.

In the second step of annotation (BlastX of tag-matching DNAs), the following preference order was used when assigning a protein ID for the tags matching different DNA datasets: the NCBI cDNA BlastX hit was used preferably compared to Ensembl cDNA hits (which are predicted cDNAs lacking verification of structure and expression), and only when no tag-matching cDNAs were available or no significant BlastX hit occurred (significance at expect value, E < 10^-5^) was genomic match used. This choice was supported by the lower BlastX annotation rate for genomic 1000-bp fragments (28.2%) compared to NCBI and Ensembl cDNAs (>70%; Additional file 1), perhaps as a result of introns, regions with low sequencing quality or errors in assembly in genomic sequences. Nevertheless, significant BlastX hits for tags matching more than one DNA dataset (223 tags) were found to have the same annotation in 186 cases (83.4%), to belong to the same protein family in 32 cases (14.4%) and to differ only in 5 cases (2.2%). These tags and those for genes selected for qPCR were subjected to careful manual annotation. Detailed annotation results can be found in Additional file 3, which summarizes all data concerning the 1339 differentially expressed tags.

In summary, we could map with high stringency 869 tags (65%), 372 to anonymous DNAs and 497 to curated Swiss-Prot protein entries (Table 2). The number of different genes identified (413) was lower than the number of tags annotated to protein, as 54 genes (13.1%) had multiple tags assigned to the same gene (average 1.2 differentially expressed tags per gene, maximum 7), suggesting they were products of sequence polymorphisms and/or alternative mRNA processing (i.e., produced by alternative splicing or use of alternative polyadenylation sites, which may affect the position of the most 3'-end *Nla*III site and thus generate different tags). In addition, 43 (8.6%) of the annotated differentially expressed tags and 37 of the genes (9%) were identified as putatively derived from natural antisense RNA transcripts, as they had an inverted match to a sense cDNA or a direct match to an antisense cDNA.

### Most up- or down-regulated tags

We first looked at the 10 transcripts that underwent the largest up- or down-regulatory change for each challenge that could be annotated to well characterized genes (Table 3, see Additional file 3 for all differentially expressed transcripts). These included transcripts for a range of calcium-binding proteins or participants in CaBP protein complexes involved in muscle contraction or cytoskeleton organization, such as *actn3*, *mle3*, *mlrs*, *tnni2*, *tpm1*, *prvb *and *telt*, which were rapidly and strongly up-regulated in gills of fish exposed to water with low [Ca^2+^]. Two genes, *entk *(involved in proteolysis) and *rl11 *(translation), were among the top 10 up-regulated transcripts at 12 h for both LowCa and HighCa, while *co8a1 *(an extracellular matrix constituent) and *frim *(iron ion oxidation and transport) were among the most down-regulated for both LowCa and HighCa, suggesting that some common mechanisms were activated.

**Table 3:** Top 10 up- and down-regulated annotated tags for each challenge

	Tag count				
				
**Tag sequence**^**1**^	Control	Treatment	**Fold change**^**2**^	Gene	Description
**Up-regulated by low calcium at 2 h:**					

TTGTTTGAGACCGATGCAGCAG	1	113	113	*tpm1*	α tropomyosin
ATGTCGGCTACCTGGTTGTAGG	0	38	110	*mle3*	Myosin light chain 3, skeletal muscle isoform
TGAAGACGAGTGGAAGGGAGGG	0	33	96	*telt*	Telethonin
GACCTGAGGTCCAACCTGAAGC	1	69	69	*tnni2*	Troponin I, fast skeletal muscle
GGATTATTTTCCAAAATAAAAA	0	23	67	*mlrs*	Myosin regulatory light chain 2, skeletal muscle isoform
AGACGGTGACCACTCTGACCCA	0	20	58	*adt1*	ADP/ATP translocase 1
TTGGAGTTCTTTACTGGAATCA	0	20	58	*actn3*	α actinin 3
TGGGCCGCCTTCCCCCAGATGT	0	17	49	*mlrs*	Myosin regulatory light chain 2, skeletal muscle isoform 2
ACGATTGTACCAGACTAAGATT	3	144	48	*prvb*	Parvalbumin β
ATTTTAGCTATTTCTCCTTGAT	0	15	43	*catz*	Cathepsin Z

**Up-regulated by low calcium at 2 h:**					

TGATGGTTTCTGAAGGTGGCGC	0	12	35	*adt2*	ADP/ATP translocase 2
CGCTGGTTCCAGCAGAAGTATG	0	10	29	*rl11*	60 S ribosomal protein L11
TCAAACTGTCTGTTTTCACCTG	0	9	26	*dnli3*	DNA ligase 3
ACGTTCCCCATCTGCCCTATTG	0	8	23	*ndk*	Nucleoside diphosphate kinase
TATTAATTTGGTTTGTTACCGT	0	8	23	*entk*	Enteropeptidase
TCATACACTGGTGGATTTGGGA	0	7	20	*rl37a*	60 S ribosomal protein L37a
CTAGCTGGCTACCAGAGGCAGG	0	7	20	*k1c13*	Keratin, type I cytoskeletal 13
GAGTTAAACAGTAGGGACAAGG	0	7	20	*zc3hf*	Zinc finger CCCH domain-containing protein 15
CAAACTAAAAGAATAAACCTCG	0	7	20	*suca*	Succinyl-CoA ligase [GDP-forming] subunit α, mitochondrial
TTCACAAACAAATCAAGTGCTT	0	6	17	*rab7a*	Ras-related protein Rab-7a

**Up-regulated by high calcium at 2 h:**					

TCATAGCTGCTCACTAAATCCA	0	69	200	*apoeb*	Apolipoprotein Eb
TATTAATTTGGTTTGTTACCGT	0	28	81	*entk*	Enteropeptidase
CGCTGGTTCCAGCAGAAGTATG	0	13	38	*rl11*	60 S ribosomal protein L11
TGCTACTCTTCGTCAGACACCC	0	9	26	*ran*	GTP-binding nuclear protein Ran
AATGAACCATAAATTGGTGCAC	0	8	23	*arpc4*	Probable actin-related protein 2/3 complex subunit 4
CCGAGTTTGTCTTATTACAAAC	0	7	20	*flcn*	Folliculin
GCATCAATGGGGTCATAATATT	0	7	20	*pttg*	Pituitary tumor-transforming gene 1 protein-interacting protein
TCCCGCCTTCACAAGCGCATTG	0	7	20	*s14l1*	SEC14-like protein 1
TTGCAGCTGATCCCTCTGATAG	0	6	17	*nmes1*	Normal mucosa of esophagus-specific gene 1 protein
GACTAATATAAAAGACCTTTTT	0	6	17	*huwe1*	E3 ubiquitin-protein ligase HUWE1 (Fragment)

**Down-regulated by low calcium at 2 h:**					

TCATACACTGGTGGATTTGGGA	21	0	61	*rl37a*	60 S ribosomal protein L37a
GGATTTGGTCTCTTTGATTAAT	15	0	43	*rla2*	60 S acidic ribosomal protein P2
GTCGTGTCTGAGCTGTGTGCCT	12	0	35	*parpt*	TCDD-inducible poly [ADP-ribose] polymerase
GACACGGACTAAAAGACACACA	11	0	32	*psb7*	Proteasome subunit β type-7
GATGCTCAGAACAACAAGTTGG	11	0	32	*frim*	Ferritin, middle subunit
CATGTATCTACGTAAAACTGAT	8	0	23	*gima7*	GTPase IMAP family member 7
GAATCGCTTAGTCGGGCATTTG	8	0	23	*ndrg1*	Protein NDRG1
AAGAAGGCCAAAGTTCTGATAC	7	0	20	*agr2*	Anterior gradient protein 2 homolog
TCCCGTGTTGTCAGAATATTAT	7	0	20	*hm20b*	SWI/SNF-related matrix-associated actin-dependent regulator of chromatin subfamily E member 1-related
TTGTTCTGTTTGTAGTCACAAG	7	0	20	*selh*	Selenoprotein H

**Down-regulated by low calcium at 12 h:**					

TTCAGGGAACTGGTGACTGCAC	47	1	47	*co8a1*	Collagen α1(VIII) chain
AACAAAATCCTGCACCCTGTTT	12	0	35	*hsp71*	Heat shock 70 kDa protein 1
ACCGACGTGTTCTGCGCAGGGA	28	1	28	*ileu*	Leukocyte elastase inhibitor
CCCTTCATCACCGAGGAGCTGT	9	0	26	*syvm*	Valyl-tRNA synthetase, mitochondrial
ACGTTTACGTCCGCAAGGTCAA	7	0	20	*rs3a*	40 S ribosomal protein S3a
GCGGAGCTGGGCATCACCGAGT	7	0	20	*tcpb*	T-complex protein 1 subunit β
TTCAAAAATGGGAGGGTGGTGG	6	0	17	*cof2*	Cofilin-2
TGGCCTGACAAGATTTTTGTTC	6	0	17	*mpeg1*	Macrophage-expressed gene 1 protein
TCCAAAATGTGAGTTCCTCTTT	6	0	17	*tp4a1*	Protein tyrosine phosphatase type IVA 1
ACGAGCGCCTGATCCGGCTGGT	6	0	17	*abcf3*	ATP-binding cassette sub-family F member 3

**Down-regulated by high calcium at 12 h:**					

TTCAGGGAACTGGTGACTGCAC	47	0	136	*co8a1*	Collagen α1 (VIII) chain
GATGCTCAGAACAACAAGTTGG	12	0	35	*frim*	Ferritin, middle subunit
CCCAGGGTGATGACCAGGGTGC	9	0	26	*horn*	Hornerin
TACTGTTTTGGATTTGTAAATA	9	0	26	*doc10*	Dedicator of cytokinesis protein 10
TCTGCTCTAACCTTATAATTCC	9	0	26	*mk12*	Mitogen-activated protein kinase 12
TGGGCCGCCTTCCCCCCAGATG	9	0	26	*mlrs*	Myosin regulatory light chain 2, skeletal muscle isoform type 2
ACGTTTACGTCCGCAAGGTCAA	7	0	20	*rs3a*	40 S ribosomal protein S3a
AAGACACATCTACAGTGGCAGT	7	0	20	*g3p*	Glyceraldehyde-3-phosphate dehydrogenase
ATGATGACGATGATTTCTTTGC	7	0	20	*igll1*	Immunoglobulin lambda-like polypeptide 1
GCTTCTTTTATCTGAGCACTGG	7	0	20	*snx19*	Sorting nexin-19

^1 ^Specific sequence of each differentially expressed 26-bp SuperSAGE tag after removal of the common CATG site. ^2 ^Fold induction or repression for up- or down-regulated genes, respectively.

### Clustering of expression patterns and enriched gene ontology

*K*-means clustering was used to group transcripts according to their patterns of expression between control and low Ca^2+ ^water over time (LowCa analysis) and across the three Ca^2+ ^availability conditions at 12 h (12 h analysis, to compare the effects of high vs. low Ca^2+ ^water challenges).

**Figure 2:**
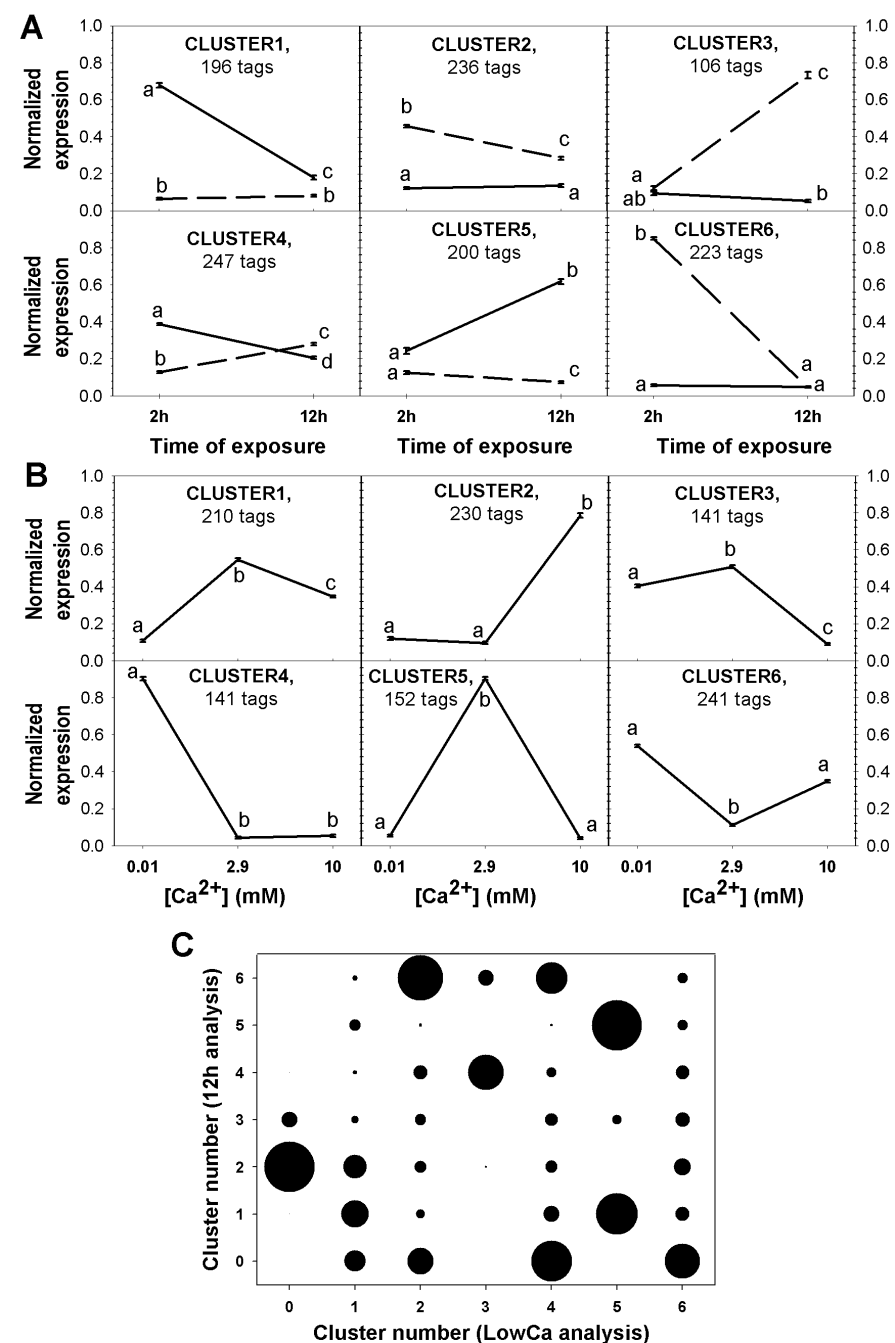
**Results from *K*-means clustering of differentially expressed SuperSAGE tags according to their expression patterns**. Pattern of expression of all tags for each cluster (mean ± S.E.M of expression, normalized by making the sum of tag counts for all the analyzed libraries equal to 100%), (A) for the LowCa analysis (effects of transfer to 0.01 mM Ca^2+ ^at 2 or 12 h), (B) for the 12 h analysis, describing tag expression at 12 h exposure to LowCa (0.01 mM Ca^2+^), control (2.9 mM Ca^2+^) or HighCa (10 mM Ca^2+^) water. The number of tags grouped in each cluster is shown in each graph. C- Bubble graph showing the relation between clusters of the two analyses. Bubble area is proportional to the number of tags in common by each pair of cluster number (LowCa and 12 h analysis). Tags falling in clusters numbered "zero" were those not analyzed (due to failure of inclusion criteria, see methods) for a given analysis (LowCa or 12 h). Solid line - control; dashed line - LowCa.

For each analysis, six clusters (optimal number of *K *= 6 obtained by Gap statistics) were obtained (Figure 2A and 2B), containing 192 ± 44 (mean ± standard deviation) tags each, which showed clear distinguishable patterns between them. Since most transcripts were common to both analyses, the number of tags common between clusters of the two analyses is also represented in Figure 2C. To understand the biological significance of the identified differentially expressed transcripts, the gene ontology [28] terms (GO) significantly enriched in the whole lists of differential expressed tags or in each of the clusters compared to the normal gill transcriptome (i.e., the annotated SuperSAGE profiles obtained in control water at 2 and/or 12 h) were identified and are listed in Additional files 4 and 5.

The biological process categories most significantly over-represented in the global lists of differentially expressed tags were mainly related to muscle contraction and cytoskeleton: categories "regulation of muscle contraction", "muscle contraction" and "cytokinesis" for both LowCa and 12 h analysis; the "positive/negative regulation of ATPAse activity" and "response to calcium ion" in the LowCa list and the "actin filament-based movement" in the 12 h list. The "phosphocreatine biosynthetic process", involved in cellular energy homeostasis, was enriched in LowCa at 2 but not in the global 12 h list. Among the enriched molecular functions in both analyses were the "structural constituent of muscle" and "motor activity categories", while the "calcium channel regulator activity" was enriched in the LowCa list; finally, enriched cellular components included "sarcomer", "myofibril" and the troponin/myosin complexes.

### Enriched categories in the "LowCa analysis"

When detailing the expression patterns of transcript tags whose expression was altered by water with low [Ca^2+^], cluster 6, grouping 223 tags with a strong up-regulation at 2 h but not at 12 h (Figure 2A), had the highest number of enriched categories (273) with the highest significance probabilities (adjusted p value down to 10^-52^) and was the main contributor to enrichment in the global list of differentially expressed transcripts (containing 89% of the enriched categories in the global LowCa list, Additional file 4). This suggests a strong coordinated response of functionally related genes in response to the LowCa stimulus at 2 h. Enriched biological processes were mainly related to Ca^2+ ^signaling or homeostasis, muscle contraction and cytoskeleton, including all the categories indicated above plus specific categories such as "calcium-mediated signaling", "calcium ion homeostasis" and "actin cytoskeleton reorganization". Transcripts grouped in this cluster and responsible for the enrichment in these categories include actin α skeletal muscle (*acts*), several myosin (*myss, mle3, myl4, mlrs*) and troponin forms (*tnnc1, tnnc2, tnni1, tnni2, tnni3, tnnt2, tnnt3*), α-tropomyosin (*tpm1*), the sarcoplasmic/endoplasmic reticulum (SR/ER) calcium ATPase 1 (*at2a1 *or *serca1*), the sarcomeric mitochondrial creatine kinase (*kcrs*) and creatine kinase muscle type 1 (*kcrm1*). Glucose metabolism also appears to have been affected in LowCa, with "gluconeogenesis" enriched in this cluster (e.g. genes *f16p2 *and *g3p*), as well as up-regulation of genes involved in glycolysis, such as *aldoa, enob *and *g3p*. This suggests an increase in the conversion of non-carbohydrate substrates (e.g. amino acids or lipids) into glucose and glucose oxidation, probably to meet the energetic needs of the acclimation response.

In addition to several molecular functions related to muscle contraction/cytoskeleton (Additional file 4), the "calcium ion binding" category was enriched, with half of the CaBPs identified grouping in this cluster. The "creatine kinase activity" (involved in cellular ATP homeostasis) was also enriched with several creatine kinase genes grouping in the cluster (*kcrb, kcrf, kcrm1, kcrs*), and enriched cellular components included sarcomer, troponin and myosin complexes, myofibril and cytoskeleton.

Changes in expression of 30% of the differentially expressed transcript tags in cluster 6 appear to be specific to the early-response to LowCa, as they did not meet the criteria for the 12 h differential expression among water types (Figure 2C).

Cluster LowCa-3 contains 106 transcript tags also up-regulated by LowCa but only after 12 h (Figure 2A). Cytoskeleton remodeling appears to be induced also at 12 h, with enrichment in "intermediate filament-based process" and up-regulation of keratin 13 (*k1c13*) and the intermediate filament protein ON3 (*ion3*), while "glutathione transferase activity" was also enriched (genes *gsta *and *mgst3*), suggesting an increase in detoxification activity. Most transcripts in this cluster (66%, e.g. *k1c13 *and *ion3*) fall into cluster 4 of the 12 h analysis (Figure 2C), indicating they were regulated by LowCa but not HighCa at the 12 h exposure, while 29% (e.g. *gsta *and *mgst3*) were also regulated by HighCa (cluster 12 h-6).

Cluster LowCa-2 includes transcripts up-regulated by LowCa at both 2 and 12 h (Figure 2A), mainly enriched in categories related to energy generation such as "mitochondrial electron transport, NADH to ubiquinone", "ATP synthesis coupled electron transport", "NADH dehydrogenase (ubiquinone) activity", "mitochondrion", and contains transcripts for several NADH dehydrogenases and NADH-ubiquinone oxidoreductases. Thirty eight percent of the cluster transcripts (including *nu2 m *and *nua4l*) showed a general up-regulation by both low and high calcium challenges (cluster 12 h-6, Figure 2).

Interestingly, clusters LowCa-1 and -5 grouped tags with changing expression levels in the control water over time (Figure 2A), but down-regulated by LowCa at 2 or 12 h, respectively. Whether or not these patterns of temporal variation in expression in control water reflect a response to stress, a natural control by circadian rhythms (our sampling times of 2 or 12 h coincided with the beginning and end of the light period, at 8 a.m. and 8 p.m., respectively) or other type of control remains to be determined. Cluster 1 was enriched in the biological processes "mitotic G2 checkpoint" and included genes related to cell division such as *ccnd2*, and "defense response" (e.g. genes *ha2b*, *hg2a*), while cluster 5 was enriched in the processes "response to unfolded protein", "sleep", "response to UV" and "positive regulation of Wnt receptor signaling pathway" (genes *hsp71*, *grp78*). Ninety percent of the transcripts in cluster LowCa-5 were down-regulated by both Low and HighCa water at 12 h and fell into clusters 1 or 5 from the 12 h analysis (Figure 2B and 2C). Finally, cluster LowCa-4 contained transcripts down-regulated by LowCa at 2 h and up-regulated at 12 h and was mainly enriched in "proteolysis" (including genes for several proteasome subunits *psa1*, *psa6*, *psa7*, *psb8 *and *prs7*; the calpain subunit *can2 *or the enteropeptidase *entk*) and "tissue regeneration" (e.g. fibulin 5, fbln5), probably representing genes involved in tissue/cell remodeling during the responses towards acclimation to external Ca^2+ ^changes.

### Enriched categories in the "12 h analysis"

The most interesting clusters for the comparison between the effects of low and high water [Ca^2+^] exposure at 12 h (Figure 2B) are clusters 5 (transcripts down-regulated by both water types) and 6 (up-regulated by both), and clusters 2 and 3 (up/down-regulated by HighCa but not LowCa) (Additional file 4). Cluster 5 is enriched in "regulation of striated muscle contraction" (genes *tnni1*, *tnnt3*) while cluster 6 is enriched in "collagen catabolic process", containing genes for proteases such as *pepd *but also one collagen form, *co8a1*. Cluster 2 includes transcripts only up-regulated by HighCa at 12 h but not by LowCa, 43% of which are also not regulated by LowCa in the 2 h exposure time (Figure 2A, B and 2C). However, no GO category was significantly enriched in this cluster, indicating that it contains a heterogeneous group of non-functionally related genes. Examples of genes only up-regulated by HighCa include ubiquitin (*ubiq*, proteolysis), the GTP-binding nuclear protein Ran (*ran*, nucleocytoplasmatic transport) and serine/threonine-protein kinase *sgk1 *(stress response and negative regulation of apoptosis).

Cluster 3, containing genes down-regulated by HighCa at 12 h but not (or only slightly) by LowCa is significantly enriched in 124 categories (Additional file 4), 66% of which were also enriched in cluster LowCa-6, such as "muscle contraction", "cytokinesis", "positive regulation of ATPAse activity", "actin filament-based movement" and "phosphocreatine biosynthetic process"/"creatine kinase activity". This result indicates a difference in the responses to low or high Ca^2+^, with the same processes that were up-regulated in LowCa at 2 h being down-regulated in HighCa at 12 h (although we do not know if they were regulated in HighCa at 2 h, since this group was not included in the study). Specific categories enriched in this cluster compared to clusters of the LowCa analysis include "protein secretion", "cell motility" or "ephrin receptor signaling pathway" (with down-regulation of the ephrin type-A receptor 2, *epha2*, and the proto oncogene tyrosine-protein kinase *yrk*).

### Differential transcript expression of proteins from selected functional groups

To focus on the effects of water Ca^2+ ^availability on the gill intracellular signaling machinery and on the gill effector mechanisms, we highlight in Table 4 five selected functional categories and related genes that were identified as differentially expressed through transcript tag annotation. Thirty genes (41 transcripts) encoded different CaBPs and mainly grouped to clusters LowCa-6 (51%) and -4 (24%), indicating that the expression of most identified CaBPs was rapidly up-regulated in response to LowCa water (e.g. calsequestrin, parvalbumin β and several myosin forms), while 19% was down-regulated by HighCa at 12 h (cluster 12 h-3, e.g. calsequestrin). One tag mapping to the important intracellular Ca^2+^-sensing and signal transduction protein calmodulin (CaM) was slightly (1.5-1.7-fold) but significantly (p < 0.05) down-regulated by LowCa at both 2 and 12 h, but is not included in our dataset of differentially expressed transcripts as its expression ratios are below our cut-off value of 2-fold.

**Table 4:** Summary list of proteins/genes belonging to selected functional groups to which differentially expressed tags were annotated

Category	Differentially expressed transcript (tag) annotation	
**Calcium binding proteins (CaBP)**	ACTN3, α actinin 3	MYL4, Myosin light chain 4 *
	AT2A1, Sarcoplasmic/endoplasmic reticulum calcium ATPase 1	MYL6, Myosin light polypeptide 6
	CAN2, Calpain-2 catalytic subunit *	PERE, Eosinophil peroxidase
	CASQ1, Calsequestrin-1	PERF, Perforin-1
	CLC4 M, C-type lectin domain family 4 member M	PRVB, Parvalbumin β *
	CPNS1, Calpain small subunit 1 *	S10A5, Protein S100-A5
	CRP, C-reactive protein	S10AD, Protein S100-A13 *
	DNSL3, Deoxyribonuclease gamma	S10I, Ictacalcin
	ELA1, Elastase-1	SCUB2, Signal peptide, CUB and EGF-like domain-containing protein 2
	ENTK, Enteropeptidase	SRCA, Sarcalumenin
	EVPL, Envoplakin	TITIN, Titin
	FBLN5, Fibulin-5	TKTL1, Transketolase-like protein 1
	HORN, Hornerin	TNNC1, Troponin C, slow skeletal and cardiac muscles
	MLE3, Myosin light chain 3, skeletal muscle isoform *	TNNC2, Troponin C, skeletal muscle
	MLRS, Myosin regulatory light chain 2, skeletal muscle isoform *	YRK, Proto-oncogene tyrosine-protein kinase Yrk

**Transcription factors (TF)**	CEBPA, CCAAT/enhancer-binding protein α	JUN, Transcription factor AP-1
	DMRT1, Doublesex- and mab-3-related transcription factor 1	JUND, Transcription factor jun-D
	EGR1, Early growth response protein 1	KLF10, Krueppel-like factor 10
	ETV6, Transcription factor ETV6	MED21, Mediator of RNA polymerase II transcription subunit 21
	FOS, Proto-oncogene protein c-fos	NDK, Nucleoside diphosphate kinase
	HM20B, SWI/SNF-related matrix-associated actin-dependent regulator of chromatin subfamily E member 1-related	USF2, Upstream stimulatory factor 2
	JAD1B, Histone demethylase JARID1B	YBOX1, Nuclease-sensitive element-binding protein 1

**Other regulators of transcription**	DNMT1, DNA (cytosine-5)-methyltransferase 1	RAN, GTP-binding nuclear protein Ran
	FUBP2, Far upstream element-binding protein 2	SMYD1, SET and MYND domain-containing protein 1
	HSBP1, Heat shock factor-binding protein 1	TNNI2, Troponin I, fast skeletal muscle
	LEP, Leptin	TRI16, Tripartite motif-containing protein 16
	MURC, Muscle-related coiled-coil protein	TXNIP, Thioredoxin-interacting protein
	NC2B, Protein Dr1	UBIQ, Ubiquitin
	PAR15, Poly [ADP-ribose] polymerase 15	ZEP1, Zinc finger protein 40

**Components of signal transduction pathways/intracellular signaling**	1433B, 14-3-3 protein β/α	MK12, Mitogen-activated protein kinase 12
	ACHA4, Neuronal acetylcholine receptor subunit α 4	P2RY8, P2Y purinoceptor 8
	APOEB, Apolipoprotein Eb	PA24C, Cytosolic phospholipase A2 gamma
	ARF4, ADP-ribosylation factor 4	PARPT, TCDD-inducible poly [ADP-ribose] polymerase
	ARHGJ, Rho guanine nucleotide exchange factor 19	PDK4, Pyruvate dehydrogenase [lipoamide] kinase isozyme 4, mitochondrial
	ARL1, ADP-ribosylation factor-like protein 1	PIN1, Peptidyl-prolyl cis-trans isomerase NIMA-interacting 1
	C42S1, CDC42 small effector protein 1	RAB7A, Ras-related protein Rab-7a
	CAP1, Adenylyl cyclase-associated protein 1	RAC2, Ras-related C3 botulinum toxin subste 2
	CEBPA, CCAAT/enhancer-binding protein α (TF)	RAN, GTP-binding nuclear protein Ran
	CLC4 M, C-type lectin domain family 4 member M	RGS1, Regulator of G-protein signaling 1
	CML1, Chemokine receptor-like 1	RGS2, Regulator of G-protein signaling 2
	CNIH, Protein cornichon homolog	RND2, Rho-related GTP-binding protein RhoN
	CO1A1, Collagen α1(I) chain	S10I, Ictacalcin
	CO1A2, Collagen α2(I) chain	SCUB2, Signal peptide, CUB and EGF-like domain-containing protein 2
	EBI2, EBV-induced G-protein coupled receptor 2 homolog	SOCS3, Suppressor of cytokine signaling 3
	EPHA2, Ephrin type-A receptor 2	STMN1, Stathmin
	FIBP, Acidic fibroblast growth factor intracellular-binding protein	TACD2, Tumor-associated calcium signal transducer 2
	FOS, Proto-oncogene protein c-fos (TF)	TITIN, Titin
	GRN, Granulins	TRI16, Tripartite motif-containing protein 16
	HG2A, HLA class II histocompatibility antigen gamma chain	UROK, Urokinase-type plasminogen activator
	HINT1, Histidine triad nucleotide-binding protein 1	VANG2, Vang-like protein 2
	IMPA1, Inositol monophosphatase	YRK, Proto-oncogene tyrosine-protein kinase Yrk
	JUN, Transcription factor AP-1 (TF)	
	KLF10, Krueppel-like factor 10 (TF)	
	MGST3, Microsomal glutathione S-transferase 3	

**Ion transporters and other proteins related to ion homeostasis or osmoregulation**	ACHA4, Neuronal acetylcholine receptor subunit α4	CLIC4, Chloride intracellular channel protein 4
	AQP3, Aquaporin-3	COX17, Cytochrome c oxidase copper chaperone
	AT2A1, Sarcoplasmic/endoplasmic reticulum calcium ATPase 1	FRIH, Ferritin, heavy subunit
	CAH1, Carbonic anhydrase 1	FRIM, Ferritin, middle subunit
	CAHZ, Carbonic anhydrase	NUDT9, ADP-ribose pyrophosphatase, mitochondrial
	CLDN28a, Claudin 28a	PLM, Phospholemman
	CLDN32a, Claudin 32a	S4A11, Sodium bicarbonate transporter-like protein 11
	CLDN38 D, Claudin 8d	SGK1, Serine/threonine-protein kinase Sgk1
	CLDN3a, Claudin 3a	VDAC2, Voltage-dependent anion-selective channel protein 2

Protein symbol and description of Swiss-Prot protein entries to which differentially expressed tags were annotated are shown. Criteria for inclusion each category were based on gene ontology annotation of Swiss-prot entries: category "Calcium-binding proteins"- genes annotated with molecular function "calcium ion binding", GO:0005509; "Transcription factors"- molecular function "transcription factor activity", GO:0003700; "Other regulators of transcription"- biological process "regulation of transcription", GO:0045449; "Components of signal transduction pathways/intracellular signaling"- genes annotated to biological process GO categories containing the words "signaling" or "signal transduction", and "Ion transporters/related to ion homeostasis or osmoregulation"- genes annotated to biological processes "ion transport", GO:0006811 or "ion homeostasis", GO:0050801. In the calcium binding protein (CaBP) category, * indicates proteins found in the CaBP database HTTP://structbio.vanderbilt.edu/cabp_database/; in the category components of signal transduction pathways, TF indicates transcription factors. For detailed information about tag sequences, counts, ratios of expression or clustering for each of these highlighted genes see Additional file 3.

Fourteen genes for transcription factors were regulated by Ca^2+ ^with a variety of induction profiles (up or down-regulation by Low or HighCa), while 14 additional regulators of transcription were identified, most of which with a rapid response to LowCa (43% in cluster LowCa-6). A large number of transcripts for components of intracellular signaling pathways were also identified (54 differentially expressed transcripts, 47 different genes) with varying expression patterns (Table 4, Additional file 3).

Eighteen genes (23 transcripts) coding for ion transporters/ion homeostasis regulators and other proteins described to influence ionic changes in gills were identified among the differentially expressed transcripts (Table 4), including five tags matching members from the claudin (CLDN) superfamily of transmembrane tight junction proteins. Annotation of these and 63 additional (non-differentially expressed) *cldn *transcript tags via Swiss-Prot BlastX mainly matched mammalian claudins 3 and 4, but it was not possible to distinguish between fish claudin paralogues, as this gene family has been largely expanded in teleost fishes [29]. *Cldn *tags were thus annotated by comparison with *F. rubripes *proteins, allowing the assignment of differential expression to fish *cldn3a*, *cldn28a*, *cldn32a *and *cldn8d *genes [following the nomenclature established in 29]. Similarly, a transcript for an aquaporin form has been annotated to fish *aqp3*, using BlastX against fish proteins only.

### Quantitative PCR of differentially expressed genes

**Figure 3:**
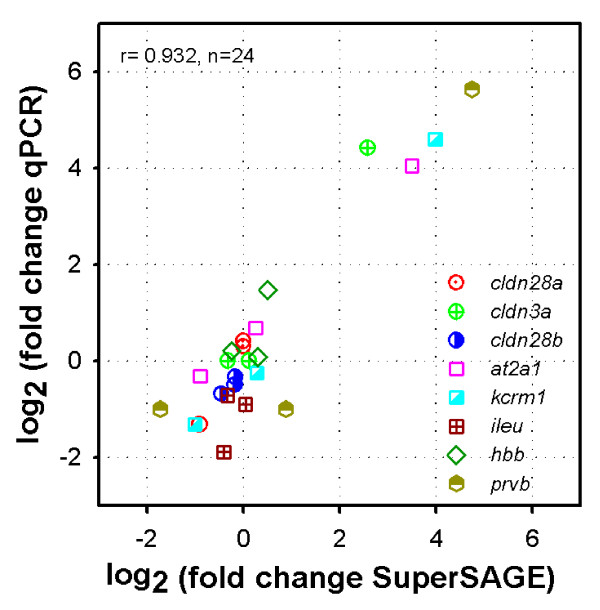
**Relationship between relative change of gene expression measured by qPCR and SuperSAGE**. Relative change of gene expression (fold change) between Ca^2+^-challenged (Low or HighCa water) and control gills for the same response period (2 or 12 h) measured by qPCR (target gene/rps18) and SuperSAGE (sum of tag counts annotating to the same gene). r is the Pearson correlation coefficient.

The same mRNAs used for SuperSAGE were individually analyzed by qPCR for eight genes detected to be differentially expressed by SuperSAGE. Gene selection took into consideration the coverage of a wide range of transcript (tag) abundance and patterns of differential expression (Additional file 6). It also included genes containing a variable number of alternative tags although qPCR primers were not designed to distinguish alternative transcripts. A statistically significant positive correlation was obtained between the SuperSAGE expression (sum of counts of all tags matching each gene) and qPCR expression levels (relative to *rps18*) for 5 of the genes tested (see Additional file 6). Furthermore, there was a highly significant positive correlation (r = 0.932, p = 3.7 × 10^-11^) for the relative expression of the 8 genes relative to control (Figure 3) between qPCR and SuperSAGE, indicating an overall concordance between the two techniques in detecting differential expression.

## Discussion

To our knowledge, this is the first genome-wide transcriptome profile of gill tissue to water Ca^2+ ^availability, and the deepest gill transcriptome study to date, identifying differential expression in 1,339 out of 79,367 different transcripts analyzed. Other studies have analyzed gill transcriptomic changes in response to different challenges such as osmotic stress, heat stress, hypoxia or infection by parasites [30-33], but the depth of analysis was much lower (maximum 16,000 different cDNA clones analyzed) and none was concerned with responses to calcium *per se*.

Three notes of caution should be considered in the interpretation of these results. First, the gill is a heterogeneous, multifunctional organ composed by several types of cells and tissues (epithelial, neural, muscular/vascular and bony/cartilaginous) [8] and the expression profiles produced can result from any calcium responsive cell type. Confirmation of the hypotheses generated by these data as being specific genes to Ca^2+ ^uptake/homeostasis requires confirmation by specific experiments, with focus on the gill epithelial MR cells, considered the primary site of active Ca^2+ ^uptake in this tissue and where most significant changes are expected to occur [3,7]. Second, gene ontology enrichment analysis is mainly based on the annotation and comparison of lists of differentially expressed genes with data that is biased towards a few (mainly mammalian) model organisms and the involvement of a particular protein in biological processes in fish may differ from that in those species [34]. Third, in common with other transcriptomic studies the produced gene sets are based on altered levels of mRNA and assume parallel, but not confirmed, changes in protein abundance [34].

Because *T. nigroviridis *does not normally live in waters containing 0.01 mM Ca^2+^, it could be questioned whether some of the transcriptomic changes reflect acute stress induced by the low [Ca^2+^] itself. This is unlikely since the "response to stress" biological function category was not enriched among the differentially expressed genes. Furthermore, of 41 proteins included in "response to stress" in the global data set only five (Serine/threonine-protein kinase Sgk1, Serine/threonine-protein kinase Sgk3, Heat shock cognate 71 kDa protein, Heat shock 70 kDa protein 1 and two isoforms of telethonin were differentially expressed, each in a different cluster and equally represented as up-regulated in LowCa at 2 h and 12 h and HighCa at 12 h (additional file 3).

### Plasma total calcium and gill expression of *ecac*

*T. nigroviridis *exposed to lower [Ca^2+^] had lower total calcium plasma levels than those exposed to high [Ca^2+^] at both 2 h and 12 h post-transfer (Figure 1), indicating a short-term dependence of total circulating levels on external [Ca^2+^], as observed in other fish species [7,35,36]. These changes are likely the result of the coordinated action of the gill, intestine and kidney, with decreased gill calcium uptake in LowCa water. This is supported by the up-regulation of the (apical side) epithelial Ca^2+ ^channel (*ecac*) mRNA detected by qPCR at 12 h (Figure 1) which suggests that this compensatory effector mechanism was activated in order to increase transepithelial Ca^2+ ^transport and restore Ca^2+ ^homeostasis. The counteracting mechanism was also observed in HighCa water with *ecac *mRNA expression down-regulated. The plasma calcium and *ecac *response to changing water calcium are similar to what has been previously described in other fish species [10-13] and acted as a positive control in our experiment.

### Global Analysis of SAGE data

With approximately 70,000 transcripts analyzed per library and assuming around 300,000 transcripts are expressed per cell [26,37], our analysis provides ~1-fold coverage for transcripts expressed at >0.9 copies per cell and thus the produced profiles are comprehensive of the gill transcriptome. The tag extraction yield was 57% and approximately 70% of the unitags were singletons. These statistics are in agreement with other reports using the same methodology [26,37].

The rate of annotation was high with 65% of the 1,339 differentially expressed SuperSAGE tags which could be annotated (57% of which matched known proteins). The specificity of tag mapping (only 2-4% tags mapped to multiple genomic locations) was also high and within the level expected for 26-bp tags [38]. The assignment of multiple tags to 13.1% of the genes annotated highlights the capacity of SAGE to detect and discriminate a diversity of mRNA transcripts [22,26,39,40]. However, for 470 tags (35%), some of which among the top 10 most-differentially expressed tags for each challenge, no DNA or protein could be assigned. These results also reflect the capacity of SAGE to identify novel transcripts with potential relevance to the physiological process in study, given the magnitude of their differential expression. These can be isolated and identified [Additional file 7, [41]] and will be an objective for future studies.

Finally, there was general concordance between SuperSAGE and qPCR in detecting differential expression of genes within a wide range of abundance levels, patterns of differential expression and variable number of alternative tags (Figure 3 and Additional file 6), as found in other SAGE studies using similar analysis [26,27,42-44]. The lack of correlation for a minority of genes tested by qPCR may have been caused by their low expression levels and presence of multiple tags in all of them, a fact that has also been previously reported in other studies [e.g.,[45-47]].

### Muscle contraction, cytoskeleton organization and cytokinesis

The main biological processes affected by short-term changes of water [Ca^2+^] were those related to muscle contraction, cytoskeleton organization and cytokinesis, with a rapid up-regulation of several actin, myosin and troponin forms, α-tropomyosin and α-actinin3, among others, 2 h after exposure to low [Ca^2+^]. Some of the same genes were down-regulated 12 h after exposure to high [Ca^2+^].

The concerted altered expression of those genes suggests that cytoskeletal modulation and/or cell proliferation are part of the process of acclimation of *T. nigroviridis *gills to changed [Ca^2+^]. Furthermore, it is possible that MR cells are key elements of these changes, since several studies have shown modifications to the MR machinery in the gill and skin epithelia in several fish species in response to manipulations of environmental [Ca^2+^] [48-52]. An important role for cytoskeletal actin in the MR cell short-term response to salinity change was previously suggested [53], since disruption of actin polymerization was able to block the rapid apical crypt closing in response to hypotonic shock, while a thick annular actin ring was detected at the opening of these crypts in MR cells and was suggested to control its opening/closure [54,55]. Although other explanations cannot be excluded, this could indicate that the transcriptomic changes we observed may be, at least to some extent, part of a common response to situations challenging ion homeostasis (such as salinity changes and specific changes in [Ca^2+^], [Na^+^] or both) in which cell number, area and morphology of gill epithelial cells (particularly the specialized ion transporting MR cells) are modified with consequent modulation of ion fluxes [e.g. [46,54,56]]. While most studies dealt with long-term acclimation periods of days or weeks, there are also reports showing MR morphological/proliferative modifications shortly after the challenge [e.g., [45-47]]. Additionally, other components of the cytoskeleton were also regulated by low or high Ca^2+ ^levels, including proteins forming the intermediate filaments (several cytokeratins and ION3) and the microtubule component β5-tubulin, consistent with a proposed role for the MR cell microtubule network in Ca^2+ ^uptake in tilapia larvae [57]. Thus, the role of the cytoskeleton and the underlying processes during the early cellular adjustments of MR cells to ion (in particular calcium) and osmotic regulation should be further investigated.

Transcriptional changes of the actin cytoskeleton components could be also linked to changes in the structure and organization of the gills. Muscle cells are present in the gill vasculature and in adductor/abductor muscles that control the angle between gill filaments [8,58] and up-regulation of cytoskeletal actin, troponin and myosin complexes, and myofibril in low calcium may reflect the key role of Ca^2+ ^in muscle contraction. Indeed, the concomitant up-regulation of cytoplasmatic CaBP PVB, creatine kinase, Ca^2+ ^pump AT2A1 and the enzyme CKM1, all described to play a role in muscle relaxation [59,60], suggest a coordinated action in muscle function and/or cytoskeleton reorganization in gill tissue.

### Calcium ion homeostasis

One of the most enriched GO categories induced by low water Ca^2+ ^availability was, as expected, calcium-ion homeostasis. A rapid up-regulation after 2 h exposure to LowCa water was detected for *AT2A1 *(*SERCA1*), the Ca^2+ ^pump responsible for re-sequestration of Ca^2+ ^into the SR/ER that, together with SR/ER Ca^2+^-release channels and cytoplasmatic/ER luminal CABPs, allow the tight regulation of cytosolic [Ca^2+^] in numerous cell types [61]. In addition, several genes for Ca^2+^-buffering CABPs were also up-regulated by LowCa water after 2 h (Table 4), including the ER lumen CaBPs calsequestrin-1 and sarcalumenin, or the cytosolic CaBPs parvalbumin β and ictacalcin [59,61], revealing for the first time a tight and rapid up-regulation of several elements of the Ca^2+ ^homeostasis control machinery at the transcriptional level in fish gills. Whether the detected changes in expression are a general cellular response of fish gill to low calcium and/or are more specifically related to the Ca^2+ ^uptake pathway to maintain body calcium balance remains to be determined. However, the current accepted model for transepithelial Ca^2+ ^uptake across fish gill MR cells includes a transcellular transport step of Ca^2+ ^bound to CaBPs, such as Ca^2+^-calmodulin, parvalbumin and ictacalcin, through the cytoplasm or its sequestration into organelles (mainly the ER) [62,63]. The up-regulation after 2 h exposure to LowCa water of a number of CaBPs previously identified as Ca^2+^-buffers supports their possible role in Ca^2+ ^uptake across fish gills, a hypothesis that may be validated by confirming these transcriptional changes specifically in the gill MR epithelial cells responsible for Ca^2+ ^uptake.

### Ca^2+ ^sensing and signal transduction

Very low and unchanging gene expression levels of calcium sensing receptor (*CaSR*), the membrane receptor which senses extracellular calcium levels, were detected by qPCR and SuperSAGE (not shown). CaSR has been previously localized to the MR cells in fish gill epithelia and shown to activate phospholipase C and mitogen-activated protein kinase (MAPK) signaling in response to external [Ca^2+^] changes [16]. Our results are consistent with reports in which the mRNA and protein expression of CaSR were unaffected by calcium constraint (in water or food) or by salinity changes [4,64].

SuperSAGE detected the differential expression of a large number of CaBPs (Table 4), including four proteins from the S-100 family (HORN, S10A5, S10AD and S10I), 75% of which were up-regulated after 2 h exposure to low water [Ca^2+^], while CaM was down-regulated at both 2 and 12 h. The immediate early gene response (induction at <2 h) response of these CaBP genes support the notion that they may be part of an intracellular Ca^2+ ^sensor network [65,66] in fish gills involved in the initial perception of Ca^2+ ^availability and in the consequent activation of signaling pathways and target proteins.

### Ion and water homeostasis

Only a small number of ion channel and transporter genes were found (Table 4) and no differential expression of transporters such as *pmca *or *ncx *was detected. This is in agreement with the theory that ECaC is the only (or main) plasma membrane Ca^2+ ^transporter regulated by environmental [Ca^2+^] in gills, while the steady state expressions of PMCA and NCX may accommodate the needs for transepithelial Ca^2+ ^transport under different [Ca^2+^] [10]. However, it is also possible that the exchange activities of these and other transporters (and not their mRNA expression levels) are modulated by [Ca^2+^] through Ca^2+ ^sensing/signaling mechanisms, such as the above mentioned activation by CaBPs or cytoskeleton reorganization. For instance, the main factors regulating PMCA activity in eukaryotes appear to be Ca^2+^-bound CaM and phosphorylation by protein kinases A/C, but recent evidence in human erythrocyte membranes also point to effects of the interaction with actin cytoskeleton elements on PMCA activity [67,68].

Additional ion homeostasis related genes were found to be significantly regulated by water Ca^2+ ^levels (Table 4), including four members of the large multi-gene family of tight-junction proteins claudins (CLDNs), which have been implicated in the maintenance of hydromineral balance across osmoregulatory epithelia of euryhaline fishes [33,69-73]. It has been suggested that a decline in their expression may be related to the reshaping of the gill epithelia of euryhaline fish upon SW-acclimation and account for its "leakier" (higher ion permeability) properties. For example, both *cldn8d *and *cldn3a *expression were reduced in a salinity-dependent manner during *T. nigroviridis *acclimation for 2 weeks to FW and SW [69,70]. In tilapia and flounder gills CLDN3 proteins were reduced 4-8 days after FW-SW transfer and increased after SW-FW transfer [71,73]. In our study a decrease in *cldn8d *gill mRNA expression was detected after 12 h exposure to low and high [Ca^2+^] water compared to control water, while the expression of *cldn3a *was rapidly up-regulated by low [Ca^2+^] at 2 h and unchanged in both low and high [Ca^2+^] water at 12 h post-transfer. As for *cldn28a *and *28b *no overall effects of FW-SW transfers were observed in salmon or tilapia [71,72], but an up-regulation of *cldn28a *was detected in SW-FW tilapia 1-7 days post-transfer [71]. In our study *cldn28b *was also unaffected by water [Ca^2+^] and the slight down-regulation of *cldn28a *detected by SuperSAGE in LowCa at 2 h was not confirmed by qPCR. Finally, differential expression was detected for two alternative tags annotated to the fish *cldn32a *isoform, one of the fish *cldn *genes whose orthologs appear to have been lost in the mammalian lineage [29], and these results provide the first evidence for regulation of its gill mRNA levels in response to water ion availability. Altogether, these observations suggest short-term alterations in paracellular epithelial permeability, the details of which are not very well known.

Two tags annotated to α carbonic anhydrases (Table 4) were up-regulated in LowCa but not HighCa, consistent with the reported up-regulation of *CAH1 *and *CAH2 *mRNA in zebra fish gills after 5-6 days exposure to soft-water [12]. Cytoplasmic carbonic anhydrases in gills may assist in apical ion uptake by providing HCO_3_^- ^for apical Cl^-^/HCO_3_^- ^exchanger or protons for apical Na^+^/H^+ ^exchanger [12]. Their up-regulation suggests an impact of Ca^2+ ^availability in the gill uptake of other ions.

Finally, one tag for the water pore *aqp3*, a protein that localizes in the basolateral membrane of MR cells and expresses at lower level in SW than FW [74,75], was down-regulated by both HighCa and LowCa challenges. This could indicate lower water permeability perhaps to minimize the potential increase in paracellular permeability evidenced in the changes in CLDNs gene expression.

### Responses to low versus high water [Ca^2+^]

**Figure 4:**
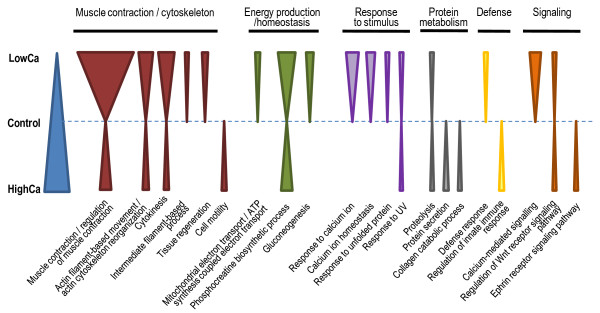
**Main biological processes affected by transfer to water at different Ca^2+ ^concentrations in *Tetraodon *gills**. Each triangle represent the differential expression of genes contained in the most represented biological processes (represented in the lower panel and grouped into broad categories in the upper panel) between LowCa (0.01 mM Ca^2+^) or HighCa (10 mM Ca^2+^) water and control water (2.9 mM Ca^2+^, blue triangle). Triangle width is inversely proportional to the adjusted p values obtained for biological process GO categories in our GO enrichment/clustering analysis (wider for most enriched processes); shown categories were selected among the most enriched GOs (adjusted p values > 10^-4^).

From the statistical analysis of differential expression, clustering and GO enrichment analysis, it became evident that the most significant changes occurred in the transfer to low [Ca^2+^] at 2 h. Whether there was an equivalent pattern of response to high [Ca^2+^] water it was not possible to confirm because it was only analyzed at 12 h. However, the analysis of this SAGE library allowed the comparison of the impacts of low vs. high water [Ca^2+^] on the gill transcriptome, summarized in Figure 4. It turned out that although specific biological processes may be regulated, the majority of the affected processes and genes respond to both challenges deviating from normal conditions of water Ca^2+ ^availability, although possibly with different time-frames, magnitudes and signal. For example, a large proportion of genes identified as up-regulated by LowCa water at 2 h were down-regulated by HighCa water at 12 h, including those more highly represented in GO terms, related to actin cytoskeleton, muscle contraction and citokinesis.

## Conclusions

We have used SuperSAGE to carry out a comprehensive analysis of the gill transcriptome during the rapid responses to changes in environmental Ca^2+^, which identified 1,339 differentially expressed transcripts. The generated transcript expression patterns provide a framework of water calcium-responsive genes in the gill during the initial response after transfer to different [Ca^2+^]. This molecular response entails initial perception of alterations, activation of signaling networks and effectors and suggests active remodeling of cytoskeletal proteins during the initial acclimation process (Figure 4). It is also possible that some alterations of gene expression may represent disruption of calcium sensitive pathways as a result of the transfer shock which we cannot distinguish at present from those of acclimation. Nevertheless, this data allows the generation of new hypotheses about the mechanisms of acclimation to environmental calcium changes, which can be tested in specific experiments (including localization studies, expression analysis at different time-frames coupled to morphological examinations of the gills, confirmation at the protein level, functional characterization, etc.) in order to refine current models of calcium transport in the gill. In addition, this study also provides a valuable diversity of novel transcripts and different transcript types which will be of great interest for further exploration.

In summary, our results indicate that when *T. nigroviridis *are transferred to a low calcium environment, their gills respond rapidly by up regulating genes related to Ca^2+ ^signaling/homeostasis but also actin cytoskeleton reorganization, cytokinesis and muscle contraction, which may be involved in previously described morphological adjustments in the cells where transepithelial Ca^2+ ^transport occurs, the MR cells, in response to altered Ca^2+ ^levels. Genes related to energy production and energy homeostasis are also up-regulated, probably reflecting the increased energetic needs of the acclimation response. The responses to high calcium availability appear to affect the same biological processes as the low calcium challenge, although with opposite effects.

## Methods

### Experimental procedure for environmental calcium exposure

All animal maintenance and handling procedures were carried out in compliance with the recommendations of the Association of Animal Behavior [76] and national legislation. Wild green spotted puffer fish, originally imported from Thailand, were purchased from a local pet shop and maintained in 200 L closed circuit aquaria in brackish water (prepared from mixing fresh water and natural sea water to 10 ppt salinity) at 26 ± 1°C, pH 7.5 ± 0.5 and natural photoperiod. They were fed once a day with frozen mollusks. After >2 months of acclimatization, in April 2007, fish were divided in three groups of six (body weight 4.4 ± 1.6 g) and transferred to 25 L aquaria containing artificial brackish water (ABW) with 10 ppt salinity and different Ca^2+ ^concentrations: 2.9 mM (control group), 0.01 mM (as in ion-poor fresh water, LowCa group) or 10 mM Ca^2+ ^(as in seawater, HighCa group). ABW (10 ppt) was prepared from deionized water and salts (Sigma-Aldrich, Madrid, Spain) with a composition based on that for artificial seawater: 131.4 mM NaCl; 2.9 mM KCl; 7.1 mM MgCl_2_; 7.1 mM MgSO_4 _and 0.01, 2.9 or 10 mM CaCl_2_, for the Control, LowCa or HighCa ABW, respectively. LowCa ABW was supplemented with 5.7 mM choline chloride (C_5_H_14_NOCl) to maintain osmolarity relatively to other ABW types, and pH adjusted to 7.5 with 1 mM NaOH in all water types. Osmolality, measured with a vapor pressure osmometer (Fiske One-Ten Osmometer, Fiske, VT, USA), was 275-300 mOsm/kg; total ammonia (NH_3_/NH_4_^+^) and nitrites (NO_2_^-^) in water, measured using TetraTest kits(Tetra Werke, Melle, Germany), were at recommended levels and temperature was kept at 26°C. No significant changes in water pH, temperature and [Ca^2+^] occurred during the time course of the experiment.

Three fish were removed from each tank after 2 h and 12 h, anesthetized (0.02%-phenoxyethanol, Sigma-Aldrich), blood sampled and fish decapitated. Blood was sampled from the cardiac dorsal vessel using heparinised glass capillary tubes and centrifuged for 5 min at 10,000 g for recovery of plasma. Complete gills (arches and filaments) were removed and snap frozen in liquid nitrogen until RNA extraction. The experiment was repeated twice in the same week (total n = 5-6 fish/group). Plasma total calcium concentration (mM) was measured using a colorimetric assay (*o*-Cresolphtalein, calcium kit, Spinreact, Girona, Spain) in duplicates, and differences between groups were analyzed by two-way ANOVA followed by Tukey' test at a statistical significance level of p < 0.05. The statistical software used was SigmaStat v.3.00 software (SPSS Inc, Chicago, USA).

### RNA extraction, construction of SuperSAGE libraries and sequencing

Total RNA was extracted from individual frozen gills with TRI Reagent (Sigma-Aldrich) and analyzed for integrity and purity by agarose gel electrophoresis and spectrophotometry, respectively. Poly(A)^+^-RNA was purified from pooled total RNA (50 μg from each fish) for each experimental group (n = 5-6 per group) using the Oligotex mRNA Mini Kit (Qiagen, Hilden, Germany) and analyzed by denaturing agarose gel electrophoresis. Five SuperSAGE libraries were constructed from 1 μg of gill poly(A)^+^-RNA each, corresponding to the groups sampled at 2 h (LowCa and control) and 12 h (LowCa, HighCa and control), following the protocol described by Matsumura et al. [23,77], but instead of ditag concatenation, cloning and sequencing, 200 ng of amplified ditags from each library (evaluated using a 2100 Bioanalyzer, Agilent Technologies, Palo Alto, California) were directly sequenced by massively parallel pyrosequencing using a GS20 sequencer (1/2 run; 454 Life Sciences, Branford, CT, USA) at the Max Planck Institute for Molecular Genetics, Berlin.

### SuperSAGE data analysis

SuperSAGE tags were extracted from sequenced ditags using the "SuperSAGE_tag_extract_pipe" program pipeline, which included stringent quality-control steps for removal of duplicated ditags, ditags containing incomplete library-identifying linkers and ditags with contaminating linker sequences in interior or ambiguous bases [24]. Comparison of tag frequencies between libraries was carried out by the "SuperSAGE_tag_freq_comp" script [24]. The SuperSAGE tags (transcripts) were deposited in NCBI's Gene Expression Omnibus [78] and are accessible through GEO Series accession number GSE19854.

The relative frequency of each tag across different SuperSAGE libraries was compared by combinations of G-tests for multiple library comparison [41,79] and Z-tests for pairwise comparisons [79,80] using the G_test and SAGEstat software (supplied by the authors) and significance levels (p) set at 0.05. Four different G-tests were performed using different subsets of libraries and decision rules [41,79]: 1) G-Test1 = Control vs. LowCa, 2) G-Test2 = pooled Control and LowCa at 2 h vs. pooled Control and LowCa at 12 h, 3) G-Test3 = Control vs. LowCa and Control vs. HighCa at 12 h, and 4) G-Test4 = Control vs. pooled LowCa and HighCa at 12 h. As a result, four different classes of differentially expressed tags were identified: 1) tags "differentially expressed between control and LowCa water independently of exposure time" (global effect) passed all decision rules in G-Test1 and the pairwise Z-test control vs. LowCa (p < 0.05); 2) tags "regulated by LowCa water over time" (time-dependent effect) passed G_intrinsic _in both G-Test1 and G-Test2, failed any of the other decision rules in G-Test1, and the control vs. LowCa effect at 2 or 12 h was confirmed by passing Z-test and failing any of the homogeneity rules in G-Test2; 3) tags "differentially expressed between control and HighCa at 12 h" passed all decision rules in G-Test3 and the Z-test for this comparison; 4) "tags with a global effect of altered water [Ca^2+^] at 12 h" passed all decision rules in G-Test4 and in the Z-test of control vs. Low or HighCa at 12 h.

In order to avoid losing biological information from low abundance transcripts by the exclusion of tags with zero counts before tag relative frequency calculation, the G-test software estimated a "zero-substitution value" of 0.345 ± 0.03 (mean ± standard error) [41,79]. Only tags whose differential expression passed statistical significance in all defined G- and Z-tests and with expression ratio R (tag counts of water [Ca^2+^]-altered libraries/tag counts of control libraries at each time point) ≥2-fold were subjected to further analysis. When tag count was zero the "zero substitution value" 0.345 was assigned before calculation of R.

### Tag-to-gene annotation

The annotation of SAGE tags (assignment to the corresponding mRNA and protein) is frequently made by interrogating databases of virtual tags extracted from unigene entries [26,27,42-44]. However, since no virtual tag database or unigene entries are available for *T. nigroviridis *an alternative 2-step annotation procedure was used. In the first step, SuperSAGE tags were mapped to *T. nigroviridis *genomic/cDNA sequences (available July 2008), running the stand alone Formatdb and BlastN scripts (blast-2.2.14-ia32-linux.tar.gz package available at NCBI [81]) against each of three DNA datasets: 1) all *T. nigroviridis *mRNA GenBank [82] ("NCBI cDNAs" dataset; 99,852 GenBank sequences); 2) transcripts resulting from known, novel and pseudo gene predictions from the *T. nigroviridis *genome [83] ("Ensembl cDNAs" dataset, 23,289 sequences); and 3) the latest assembly of the *T. nigroviridis *genome [84] ("*Tetraodon *genome" dataset, 21 chromosomes). The "SuperSAGE_tag_BLAST" suite of programs [24] were used to parse BlastN results under stringent conditions (only perfect tag-to-DNA matches, with 26/26 identical nucleotides, were accepted) and to extract DNA fragments 1000-bp upstream from tag-genomic matches.

In the second step, top BlastN cDNA/genomic matches for each tag were compared to the Swiss-Prot protein database (downloaded March 2009 [85]) using stand-alone BlastX. To assign protein identities to each tag, the expect value (E) was set at <10^-5 ^and the hierarchical preference order for BlastX matches was: NCBI cDNAs > Ensembl cDNAs > Tetraodon genome 1000-bp fragments. This process was verified manually in cases of multiple BlastX matches using queries from different DNA datasets and for the genes analyzed by qPCR. The alignment directions of BlastN (tag to DNA) and BlastX (DNA to Swiss-Prot protein) were compared to identify tags potentially derived from antisense transcripts (those presenting an inverted match to a sense cDNA or a direct match to an antisense cDNA). Tags matching members from the mammalian claudin family of proteins were assigned to their fish claudin isoform by stand-alone BlastX against the 56 *Takifugu rubripes *claudin proteins (GenBank accession numbers AAT64039-AAT64094).

### Clustering analysis

The 1,339 tags identified as differentially expressed were grouped according to their expression patterns among different libraries by *K*-means clustering analysis, using the SAGE data analysis tool and the *TransChisq *algorithm [86]. Two clustering analyses were performed: the "LowCa analysis" clustered 1,208 tags according to their pattern of expression among control and LowCa libraries (all differentially expressed tags excluding those with significant effect only for HighCa challenge and those with zero tag count in all the control and LowCa libraries), while the "12 h analysis" clustered 1,098 tags (all differentially expressed tags except those with zero tag count in all the 12 h libraries) according to their pattern of expression among 12 h libraries (control, LowCa and HighCa). The optimal number of clusters *K *was obtained by a combination of the *TransChisq *algorithm and Gap statistics run in MatLab (MathWorks Ltd., Cambridge, UK) as described in [86].

### Gene ontology (GO) annotation and enrichment analysis

Gene ontology mapping was performed on differentially expressed tags by retrieving the GO terms [28] associated with all significant BlastX hits (E value < 10^-10^) of each tag-matching DNA against the Swiss-Prot protein database. GO enrichment was then determined for each list of annotated differentially expressed tags (all LowCa-regulated tags, each of the six LowCa clusters, all 12 h-regulated tags or each of the six 12 h clusters) using a one-tailed proportion test [87] at a false discovery rate adjusted p-value of 0.01, between the number of tags containing a given GO in each list compared to the number of tags containing that GO in the normal gill transcriptome at the corresponding time point (all tags contained in control libraries at 2 and 12 h, for the LowCa analysis, or at 12 h only for the 12 h analysis).

### Quantitative polymerase chain reaction

Eight differentially expressed genes identified by SuperSAGE (*ecac*, *cldn3a, cldn28a, cldn28b, prvb, ileu, at2a1, hbb *and *ckm1*) were selected and analyzed by quantitative real-time PCR (qPCR) in the same individual RNAs used for SuperSAGE library construction. Specific primers for each gene were designed based on available genomic or cDNA sequences and on the alignment with related genes to avoid cross-amplification, using Beacon Design software (Premier Biosoft Int., Palo Alto, CA). Primer sequences, amplicon sizes and optimized temperatures used in qPCR for each pair are shown in Additional file 8. For cDNA synthesis, 4 μg total RNA was treated with DNA-free Kit (Ambion, UK) and cDNA synthesis was carried out using 500 ng of DNAse-treated total RNA, 200 ng of random hexamers (GE Healthcare, Little Chalfont, UK), 40U of MMLV reverse transcriptase (RT) (Promega, Southampton, UK) and 5U of RNAguard RNase inhibitor (GE Healthcare) in a total volume of 20 μl. Quantification was performed in duplicate reactions using SYBRgreen chemistry (Power SYBR^® ^Green PCR Master Mix, Applied Biosystems, UK) and the relative standard curve method [88], using a Bio-Rad iClycler iQ5 qPCR thermocycler and software (Bio-Rad Laboratories). PCR cycling conditions were 10 min at 95°C, followed by 55 cycles of 10 sec at 95°C, 20 sec at 60°C and 30 seconds at 72°C. A final melting curve was carried out between 60 and 98°C for all genes and showed single product/dissociation curves. All amplicons were sequenced to confirm specificity.

Standard curves relating initial template quantity to amplification cycle were generated using serial dilutions of linearized plasmid DNA containing the gene of interest, and efficiency ranged between 75-100% with R^2 ^> 0.99. No cross-amplification was detected between closely related genes (*cldn28a *and *cldn28b*) by testing the PCR over the heterologous template. Absence of DNA contamination was confirmed by testing the amplification of all genes on a sample that did not receive reverse transcriptase. The expression of three candidate reference genes (*18s, g3p *and *rps18*) was quantified in the same conditions and no statistically significant differences were found between experimental groups for any of them. *rps18 *was chosen as endogenous reference gene to normalize qPCR data as it had the lowest variation and a level of expression of the same order of magnitude to target genes. Statistical significance of relative gene expression between groups was analyzed by two-way ANOVA. Pearson correlations between the qPCR data (average expression for each experimental group) and the cumulative signal obtained by SuperSAGE (sum of tag counts annotated to the same gene) were calculated for each gene after log2 transformation of both variables. A Pearson correlation was also calculated between qPCR and SuperSAGE log2 transformed gene expression fold changes between treated groups and control at each time point. The significance level was 5%.

## Abbreviations

18S: 18S ribosomal RNA; ABW: artificial brackish water; AT2A1 (SERCA1): Ca^2+^-transporting ATPase sarcoplasmic reticulum type, fast twitch skeletal muscle isoform or sarcoplasmic/endoplasmatic reticulum Ca^2+ ^ATPase1 (alternative name); C2 h/C12 h: exposure to control (2.9 mM Ca^2+^) water for 2 or 12 h, respectively; Ca^2+^: calcium ion; CaBP: calcium-binding protein; CaM: calmodulin; CaSR: calcium-sensing receptor; CLDN: claudin; EST: expressed sequenced tag; FW/SW: fresh water/seawater; G3P: glyceraldehyde-3-phosphate dehydrogenase; GO: gene ontology; HBB: hemoglobin β; HighCa12 h: exposure to water with high (10 mM) Ca^2+ ^concentration for 12 h; ILEU: leukocyte elastase inhibitor; KCRM1: creatine kinase muscle type 1; LowCa2 h/LowCa12 h: exposure to water with low (0.01 mM) Ca^2+ ^concentration for 2 or 12 h, respectively; MR cells: mitochondrion-rich cells; NCX: Na^+^/Ca^2+^-exchanger; PMCA: plasma membrane Ca^2+^-ATPase; PRVB: parvalbumin b; qPCR: quantitative real-time PCR; RPS18: ribosomal protein S18; S.E.M.: standard error of the mean; SAGE: serial analysis of gene expression; SNP: single nucleotide polymorphism; SR/ER: sarcoplasmic/endoplasmic reticulum; TRPV6 (ECaC): transient receptor potential cation channel subfamily V member 6 or epithelial calcium channel (alternative name).

Gene/protein names and symbols are written, respectively, in italic or in plain type, and follow the nomenclature defined by Swiss-Prot [89]; in some cases, an alternative name/symbol is also indicated to facilitate comparison with available bibliography. For abbreviations of genes identified by SuperSAGE, see Additional file 3 or [89].

## Authors' contributions

PISP was involved in all aspects of the project, including experimental design and set-up, SuperSAGE libraries construction and data analysis, qPCR and writing. HM assisted in SuperSAGE libraries construction and data analysis and RT on SuperSAGE data analysis. RR was responsible for the 454 sequencing. MAST performed the GO enrichment analysis. DMP participated in the discussion of results and writing of the manuscript. AVMC devised the study, provided resources, participated in the planning of all experiments, statistical analysis, discussion of results, and writing of the manuscript. All authors read and approved the final manuscript.

## Supplementary Material

Additional file 1 **Venn diagram summarizing the mapping of differentially expressed tag sequences to the three available datasets containing *T. nigroviridis *DNA sequences**. Click here for file

Additional file 2 **Percentage of tag mapping to different *T. nigroviridis *DNA datasets using different levels of stringency**. Click here for file

Additional file 3 **Main data file summarizing all the expression (tag counts, expression ratios and clustering) and annotation data (BlastN and BlastX results) for the 1,339 differentially expressed tags identified**. Tags are sorted by LowCa cluster number, annotation and gene symbol. Significant BlastN hits (26/26 identical nucleotides) and BlastX hits (E < 10^-5^) shown in white, non-significant hits in grey. * indicates BlastX hits confirmed by manual annotation. Click here for file

Additional file 4 **List of enriched GO categories in clusters of the LowCa analysis**. Cluster number, GO code, GO type (P = biological process, F = molecular function, C = cellular compartment), description and adjusted p value are shown for significantly enriched categories (adjusted p value < 0.01). Click here for file

Additional file 5 **List of enriched GO categories in clusters of the 12 h analysis**. Cluster number, GO code, GO type (P = biological process, F = molecular function, C = cellular compartment), description and adjusted p value are shown for significantly enriched categories (adjusted p value < 0.01). Click here for file

Additional file 6 **Detailed information for the differentially expressed tags/genes analyzed by qPCR**. Includes gene accession numbers, name and sequence; tag sequence, counts, classification as differentially expressed (p < 0.05) or not, clustering and Pearson correlation coefficient and p value between SuperSAGE and qPCR results. Click here for file

Additional file 7 Parameters used in G-test statistical comparison of tag-proportions among Tetraodon gill SuperSAGE libraries Click here for file

Additional file 8 **Primer sequences and PCR product sizes of genes selected for qPCR**. Click here for file
