# Supplementary material for: Gill transcriptome response to changes in environmental calcium in the green spotted puffer fish
Source: BMC Genomics. 2010 Aug 17;11:476. doi: 10.1186/1471-2164-11-476 (PMC3091672; doi:10.1186/1471-2164-11-476)
Supplement: Additional file 1 — Venn diagram summarizing the mapping of differentially expressed tag sequences to the three available datasets containing T. nigroviridis DNA sequences. [file 1471-2164-11-476-S1.PDF]

Pinto *et al*, Additional file S1

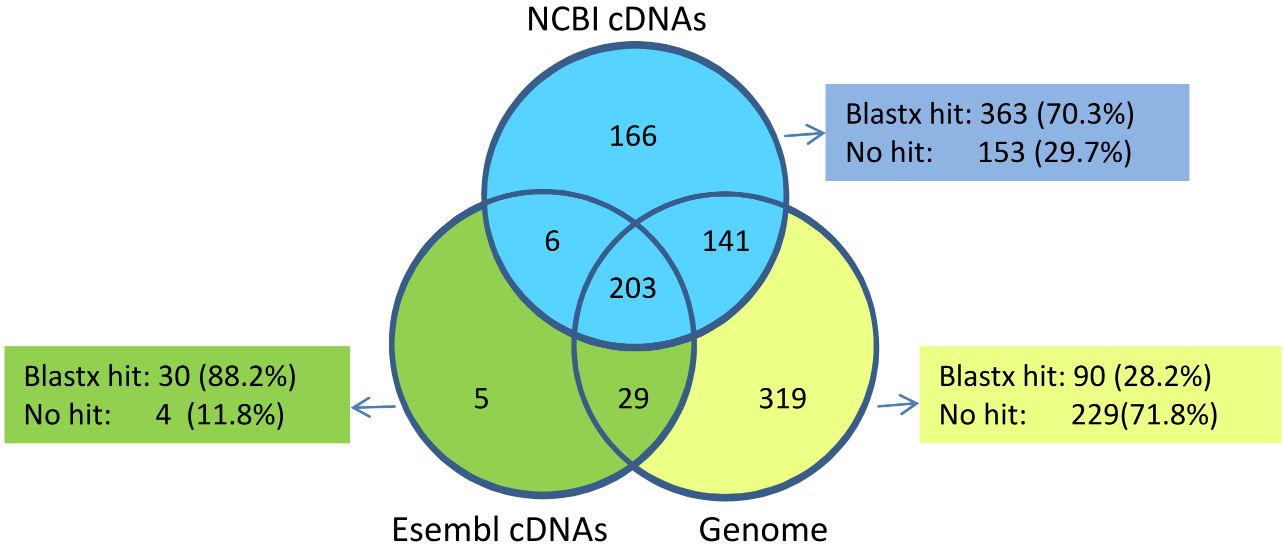

Additional figure S1. Venn diagram summarizing the mapping of differentially expressed tag sequences to the three different available datasets containing *T. nigroviridis* DNA sequences. Numbers inside each area (non-proportional) indicate the number of common or specific tags showing perfect matches (26/26 identical nucleotides) to NCBI cDNAs, Ensembl cDNAs or to the *T. nigroviridis* genome, while blocks indicate the number and percentage (in brackets) of these DNA sequences that significantly matched (E value $< 10^{-5}$) Swiss-Prot protein entries by BlastX (2-step procedure for tag annotation). Colors indicate the preference order given to the BlastX results of DNA matches from the different datasets when annotating the tags: matches to NCBI cDNAs (blue) used preferably, followed by Ensembl cDNAs (green) and genomic matches (yellow).
