# Supplementary material for: Gill transcriptome response to changes in environmental calcium in the green spotted puffer fish
Source: BMC Genomics. 2010 Aug 17;11:476. doi: 10.1186/1471-2164-11-476 (PMC3091672; doi:10.1186/1471-2164-11-476)
Supplement: Additional file 2 — Percentage of tag mapping to different T. nigroviridis DNA datasets using different levels of stringency. [file 1471-2164-11-476-S2.PDF]

Pinto *et al*, Additional file S2

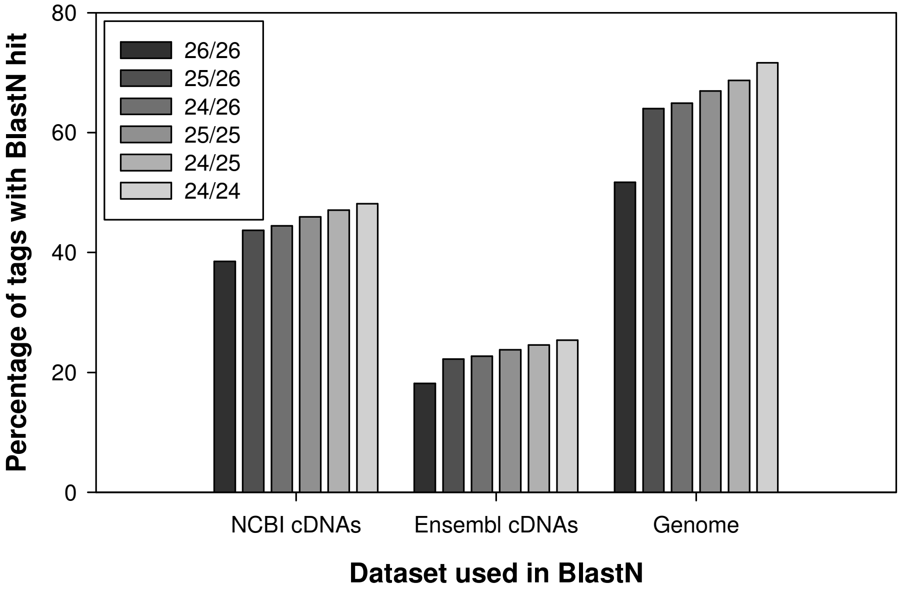

Additional figure S2. Tag mapping to different *T. nigroviridis* DNA datasets using different levels of stringency. The percentage of unique tags (out of 1,339 differentially expressed tags) with a significant BlastN hit, according to the different stringency levels indicated in the legend (identical nucleotides / aligned nucleotides), is shown for each DNA dataset.
