# Supplementary material for: Gill transcriptome response to changes in environmental calcium in the green spotted puffer fish
Source: BMC Genomics. 2010 Aug 17;11:476. doi: 10.1186/1471-2164-11-476 (PMC3091672; doi:10.1186/1471-2164-11-476)
Supplement: Additional file 7 — Parameters used in G-test statistical comparison of tag-proportions among Tetraodon gill SuperSAGE libraries [file 1471-2164-11-476-S7.PDF]

Pinto *et al*, Additional file S7

Table S7- Parameters used in G-test statistical comparison of tag-proportions among *Tetraodon* gill SuperSAGE libraries.

	Test1	Test2	Test3	Test4
**Standard subset** [1]	(C2h and C12h)	(C2h and LowCa2h)	C12h	C12h
	versus	*versus*	*versus*	*versus*
**Test subset (s)** [1]	(LowCa2h and LowCa12h)	(C12h and LowCa12h)	HighCa12h or LowCa12h	(HighCa12h and LowCa12h)
**Rule 1** [2]	$G_{intrinsic}$ ($p<0.05$)	$G_{intrinsic}$ ($p<0.05$)	$G_{intrinsic}$ ($p<0.05$)	$G_{intrinsic}$ ($p<0.05$)
**Rule 2 (homogeneity)** [2]	$G_{within,standard}$ ($p>0.05$)	$G_{within,standard}$ ($p>0.05$)	$G_{within,standard}$ ($p>0.05$)	$G_{within,standard}$ ($p>0.05$)
**Rule 3(homogeneity)** [2]	$G_{within, test}$ ($p>0.05$)	$G_{within, test}$ ($p>0.05$)	$G_{individual,test->standard}$ ($p<0.05$)	$G_{within, test}$ ($p>0.05$)
**Rule 4** [2]	$G_{pooled,test->standard}$ ($p<0.05$)	$G_{pooled,test->standard}$ ($p<0.05$)		$G_{pooled,test->standard}$ ($p<0.05$)

Parameters used in G-test statistics were: [1] chosen subsets of SuperSAGE libraries compared in each test: gill libraries from fish exposed to control water for 2 or 12h (C2h or C12h), 0.01mM Ca^{2+} water for 2 or 12h (LowCa2h or LowCa12h) and 10mM Ca^{2+} water for 12h (HighCa12h); [2] G-test statisitics decision rules and significance levels (p) for each test. For more details on G-test principles and decision rules see Schaaf *et al.* (2005) and Schaaf *et al.* (2008).

References:

Schaaf, G. J., et al., 2005. Full transcriptome analysis of rhabdomyosarcoma, normal, and fetal skeletal muscle: statistical comparison of multiple SAGE libraries. FASEB J. 19**,** 404-6.

Schaaf, G. J., et al., Statistical comparison of two or more SAGE libraries: one tag at a time. In: K. L. Nielsen, (Ed.), Serial Analysis of Gene Expression (SAGE). Humana Press, Totowa, New Jersey, 2008, pp. 151-68.
