# Supplementary material for: Gill transcriptome response to changes in environmental calcium in the green spotted puffer fish
Source: BMC Genomics. 2010 Aug 17;11:476. doi: 10.1186/1471-2164-11-476 (PMC3091672; doi:10.1186/1471-2164-11-476)
Supplement: Additional file 8 — Primer sequences and PCR product sizes of genes selected for qPCR. [file 1471-2164-11-476-S8.PDF]

# Pinto *et al*, Additional file S8

**Table S8- Primer sequences and PCR product sizes of genes selected for qPCR.**

| Gene | Primer sequence [1] | bp [2] | Ta (ºC) [3] |
|---|---|---|---|
| *trpv6(ecac)* | Fw: 5’ GCCCTTTGACTTCCTACCTGTATGAC 3’ | 112 | 60 |
| | Rv: 5’ AGTATTCCTCTCGCCTCTTTCTGC 3’ | | |
| *cldn3a* | Fw: 5’ GCTGCCGCCCTCCTGATTC 3’ | 144 | 58 |
| | Rv: 5’ TCAAACATAGTCCTTCCTCTCCAG 3’ | | |
| *cldn28a* | Fw: 5’ GCCATCATTGTTGGAGTCGC 3’ | 138 | 58 |
| | Rv: 5’ GACCAGAACTCCGGCACAA 3’ | | |
| *cldn28b* | Fw: 5’ GGACATTGTCGGAGTGGTCGT 3’ | 173 | 60 |
| | Rv: 5’ GGTGGGGTTGTAGAAGTCCTG 3’ | | |
| *prvb* | Fw: 5’ CTCTTCAACTGTTCACATCACTCTCC 3’ | 79 | 57 |
| | Rv: 5’ GGCACAAGTCATCCAATCATCATATAATC 3’ | | |
| *at2a1(serca1)* | Fw: 5’ GCTGCCTGGTGGTTCCTGTATG 3’ | 97 | 59 |
| | Rv: 5’ CAAAGTCCTCGTTCTCGTCGTG 3’ | | |
| *kcrm1* | Fw: 5’ ATTCCCGCCCAGAAGTAAAGAGG 3’ | 120 | 59 |
| | Rv: 5’ AGACGGAAAGAGCAGGTTAGC 3’ | | |
| *Ileu* | Fw: 5’ TCCGCATTCTGAGCCAAGG 3’ | 192 | 59 |
| | Rv: 5’ GGGAGTTGATTTCACCGTTG 3’ | | |
| *hbb* | Fw: 5’ CGACAACATCTTCTCCAACCTG 3’ | 97 | 57 |
| | Rv: 5’ GCTGAAGTATCTCTGGGTCC 3’ | | |
| *18s* | Fw: 5’ TGACGGAAGGGCACCACCAG 3’ | 158 | 60 |
| | Rv: 5’ AATCGCTCCACCAACTAAGAACGG 3’ | | |
| *g3p* | Fw: 5’ CTGCCGTCTGTCCAAGCC 3’ | 149 | 60 |
| | Rv: 5’ CAAAGATGGAGGAGTGAGAGTC 3’ | | |
| *rps18* | Fw: 5’ TCAAGATGTCTCTGGTCATTCCTG 3’ | 105 | 59 |
| | Rv: 5’ TGATGGCTGTGATGGCAAAG 3’ | | |

[1] Sequences of forward (Fw) and reverse (Rv) primers (5’ to 3’ sense) for all genes quantified by quantitative PCR (see abbreviations list for gene symbols), [2] amplicon sizes (bp) and [3] optimized annealing temperatures (Ta).
